# High Prevalence of Severe Hepatic Fibrosis in Type 2 Diabetic Outpatients Screened for Non-Alcoholic Fatty Liver Disease

**DOI:** 10.3390/jcm12082858

**Published:** 2023-04-13

**Authors:** Clelia Asero, Annalisa Giandalia, Irene Cacciola, Carmela Morace, Giuseppe Lorello, Amalia Rita Caspanello, Angela Alibrandi, Giovanni Squadrito, Giuseppina T. Russo

**Affiliations:** 1 Department of Clinical and Experimental Medicine, University of Messina, 98124 Messina, Italy; clelia.asero@gmail.com (C.A.);; 2 Medicine and Hepatology Unit, University Hospital of Messina, 98124 Messina, Italy; 3 Internal Medicine and Diabetology Unit, University Hospital of Messina, 98124 Messina, Italy; 4 Unit of Statistical and Mathematical Sciences, Department of Economics, University of Messina, 98122 Messina, Italy; 5 Internal Medicine Unit, University Hospital of Messina, 98124 Messina, Italy

**Keywords:** type 2 diabetes, hepatic fibrosis, NAFLD, CAP, FIB-4

## Abstract

Background: Non-alcoholic fatty liver disease (NAFLD) is a highly frequent condition in patients with type 2 diabetes (T2D), but the identification of subjects at higher risk of developing the more severe forms remains elusive in clinical practice. The aim of this study was to evaluate the occurrence and severity of liver fibrosis and its predictive factors in T2D outpatients without a known history of chronic liver disease by using recommended non-invasive methods. Methods: Consecutive T2D outpatients underwent a set of measurements of clinical and laboratory parameters, FIB-4 score (Fibrosis-4 index), and liver stiffness with controlled attenuation-parameter (CAP) performed by transient elastography (FibroScan) after excluding previous causes of liver disease. Results: Among the 205 T2D outpatients enrolled in the study (median age: 64 years, diabetes duration: 11 years, HbA1c: 7.4%, and BMI: 29.6 kg/m^2^), 54% had high ALT and/or AST levels, 15.6% had liver stiffness value > 10.1 kPa (severe fibrosis), 55.1% had CAP values > 290 dB/m (severe steatosis), and FIB-4 score was >2 in 11.2% of subjects (>2.67 in 15 subjects). Moreover, 49 (23.9%) T2D patients had clinically meaningful liver harm, with either a FIB-4 score > 2 and/or FibroScan > 10.1 kPa. At regression analysis, BMI, HbA1c, creatinine, and triglycerides values were independent predictors of liver fibrosis. Conclusions: Liver fibrosis is a frequent finding in T2D outpatients without a known history of liver disease, especially in those with obesity, hypertriglyceridemia, worse glycemic control, and high creatinine levels.

## 1. Introduction

The term non-alcoholic fatty liver disease (NAFLD) identifies a broad spectrum of clinical conditions ranging from hepatic steatosis to non-alcoholic steatohepatitis (NASH) [1]. Its prevalence in the general population is about 25% [2], and, in recent decades, it has become a major cause of end-stage liver disease, including hepatocarcinoma (HCC) and liver transplantation [3].

NAFLD is so frequently associated with extra-hepatic metabolic disorders, including obesity and type 2 diabetes (T2D) [4], that it has been termed MAFLD (metabolic-associated fatty liver disease) [5]. Thus, NAFLD can be diagnosed in up to about 70–80% of T2D patients, although its presentation, clinical course, and outcomes are quite heterogeneous.

The coexistence of NAFLD in patients with T2D has deleterious consequences for the course of both conditions. In studies on T2D patients, NAFLD has been associated with poorer glycemic control, macrovascular damage, and increased cardiovascular risk, while its relationship with microvascular complications is still poorly defined [6,7,8,9].

NAFLD can also lead to liver fibrosis, cirrhosis and hepatocellular carcinoma [10], with an overall increase in mortality rate, especially in T2D [11,12]. In fact, T2D patients may develop more severe forms of liver disease [13] and, among them, advanced stages of liver fibrosis (grades F3/F4) lead to a 10 times higher HCC risk than in the general population [14], with ~1/3 of patients developing NAFLD-related HCC even without any previous evidence of hepatic cirrhosis. Furthermore, the fibrosis stage is considered the major predictive factor for mortality for both cardiovascular disease and liver damage in these patients [13].

The high prevalence and the tendency to develop more severe forms, as well as the risks associated with NAFLD in T2D patients, make timely identification of this condition necessary in the routine care of T2D patients, as advocated by current Guidelines [15]. However, the systematic screening for NAFLD in T2D subjects is still inadequate in everyday clinical practice because of the inapplicability of liver biopsy, which is still the gold standard for the diagnosis and staging of this serious disease.

To overcome this difficulty, the use of non-invasive methods [e.g., liver ultrasound (US) [16], controlled attenuation parameter (CAP), Magnetic Resonance Imaging (MRI) based proton density fat fraction [17], and NAFLD fibrosis score (NFS)] has increasingly been used in clinical practice, as recommended by dedicated Guidelines. Thus, several scores [18], such as FIB-4 (Fibrosis-4 index), APRI (AST to platelet ratio index), FibroTest, Forns index, HepaScore, FibroMeter, NAFLD score, FLI (Ultrasonographic fatty liver indicator), and HIS (hepatic steatosis index) [19] have been developed and validated in order to identify at-risk patients and to address them to further specialistic diagnostic/therapeutic steps.

Recently, national and international guidelines have recommended a dedicated algorithm for the screening of NAFLD in T2D patients by using cheap and non-invasive tests [20,21]. Furthermore, the combined use of non-invasive methods, such as clinical scores, US, and liver elastography performed by FibroScan with CAP, is advocated to implement the identification of the highest-risk patients [22].

Despite all this evidence and the burden of the more advanced stages of NAFLD, in the clinical setting of diabetes centers, many diagnoses are under-reported, and the identification of T2D subjects at higher risk of fibrosis remains an area of uncertainty.

Accordingly, the aim of this current study was to evaluate the occurrence and severity of liver damage and its risk factors in T2D outpatients without a known history of chronic liver disease by using non-invasive methods.

## 2. Patients and Methods

All patients with T2D consecutively attending the Diabetes Unit of University Hospital of Messina from 1 January to 30 March 2021, who have consented to participate, were enrolled in this single-center study.

Exclusion criteria were any previous diagnosis of liver disease (viral hepatitis, autoimmune hepatitis, hemochromatosis, primary biliary cholangitis, Wilson’s disease, sclerosing cholangitis, biliary obstruction, and alpha-1 antitrypsin deficiency), including viral infection (HBV and/or HCV) and/or alcohol use disorder. Alcohol intake [23,24] was evaluated and quantified as the number of units of drinks per day [one drink or alcoholic unit (AU) = 12.5 g of pure ethanol contained in a glass of wine, a pint of beer, or a mini glass of spirits].

Moreover, in order to exclude cases of unknown viral infection, all participants underwent viral infection (HBV and/or HCV) markers measurements before entering the study: Australian antigen (HBsAg) and antibodies (HbsAb), Hepatitis B e-Antigen (HbeAg) and antibodies (HbeAb), and Hepatitis B core antibody (HBcAb) were dosed to exclude Hepatitis B virus infection, while anti-HCV serology was performed to rule out Hepatitis C virus infection. Further exclusion criteria were the following conditions: malignancies, heart failure (New York Heart Association class III-IV), pregnancy, end-stage renal disease, and the use of steatogenic drugs (e.g., estrogens, amiodarone, steroids, and tamoxifen).

The following clinical and laboratory data were measured and recorded in a digitalized dataset: demographic data (age and sex), body mass index (BMI) [25], presence of comorbidities (arterial hypertension and dyslipidemia), data on diabetic disease (duration, hypoglycemic treatments, number of diabetes medications, diabetes micro- and macro-vascular complications), as well as the following laboratory parameters: fasting plasma glucose (FBG), glycated hemoglobin (HbA1c), serum alanine aminotransferase (ALT), aspartate aminotransferase (AST) [26], gamma glutamyl transpeptidase (GGT), alkaline phosphatase (ALP), bilirubin, total cholesterol, HDL-cholesterol, triglycerides, albumin and gamma-globulin, red blood cells (RBC), white blood cells (WBC), platelets (PLT); values of hemoglobin (Hb), creatinine, and international normalized ratio (INR). LDL-cholesterol was calculated by the Friedewald formula.

Written informed consent was obtained from all patients, and the Institutional Review Board of district of Messina approved the study (protocol number 30/20).

## 3. Assessment of Chronic Diabetes Complications

Both micro- and macro-vascular complications of T2D were screened according to national and international diabetes guidelines [27,28].

Macrovascular disease: coronary heart disease was defined based on clinical documentation and of the reports of cardiologist specialists and/or hospital discharge (myocardial infarction, chronic ischemic heart disease, coronary heart by-pass, coronary angioplasty); a standard electrocardiogram and a cardiologist visit are performed annually in all T2D patients as part of the usual screening program. Cerebrovascular disease and peripheral arterial disease were assessed by color-Doppler ultrasonography by B-mode real-time ultrasound, as part of the periodic screening of macrovascular complications.

Microvascular disease: diabetic retinopathy was diagnosed based on direct ophthalmoscopy performed by an expert ophthalmologist and/or by fluorescein angiography within 1 year before the start of study. Diabetic kidney disease was assessed according to albuminuria measurement and estimation of Glomerular Filtration Rate (eGFR) by CKD-EPI formula [29].

## 4. Assessment of NAFLD/Liver Fibrosis

All T2D study subjects underwent evaluation of NAFLD and liver fibrosis through non-invasive methods, e.g., the measurements of serum ALT/AST and γ-GT, the calculation of FIB-4 score and the evaluation of liver stiffness through liver elastography, performed by FibroScan with controlled attenuation-parameter (CAP), for the quantification of intrahepatic fat [30,31]. Aminotransferase normal value and γ-GT were measured by standard laboratory methods; normal range of our laboratory was for serum AST and ALT values ≤ 40 UI/L and γ-GT values ≤ 50 UI/L. FIB-4 score was calculated to detect NAFLD by non-invasive scores before elastography (as indicated in Table 1): FIB-4 score values < 1.3 were considered not predictive of fibrosis, whereas FIB-4 score values > 2 were considered positive predictive of fibrosis, and FIB-4 score > 2.67 was considered a high positive predictive value of advanced fibrosis [32]. Liver Stiffness and CAP were measured by Echosens FibroScan 630 Expert, using M or XL probe. Liver stiffness values were considered as both categorical and binomial variables considering the cut-off of 10.1 kPa for severe fibrosis [33]. Liver CAP values were considered categorical variables, values < 238 dB/m were considered normal and severe steatosis values > 290 dB/m [34]. According to current guidelines [clinical practice guideline of the Italian Association for the Study of the Liver (AISF), the Italian Society of Diabetology (SID), and the Italian Society of Obesity (SIO), 2021], NAFLD diagnosis was assumed with a FIB-4 value > 2 and a liver stiffness > 10.1 kPa [13]. Three grades, from S1 to S3, were considered in order to define the severity of liver steatosis.

## 5. Statistical Analysis

The numerical data were expressed as median and range (minimum and maximum), and the categorical variables as number and percentage. The non-parametric approach was used because most of the numerical variables were not normally distributed, such as those verified by Kolmogorov–Smirnov test. Univariate and multivariate logistic regression models were estimated in order to identify significant predictive factors of hepatic fibrosis. Univariate logistic regression models were applied to the 3 independent variants FIB-4 > 2, CAP > 290 dB/m and liver Stiffness (FibroScan) > 10.1 kPa. Considering liver Stiffness (FibroScan >10.1 kPa), significative results were achieved for BMI (*p* value 0.000), HbA1c value (*p* value 0.002), and creatinine clearance value (*p* value 0.011): these results were confirmed by multivariate logistic regression models except for the creatinine clearance value (*p* value 0.779). The same univariate regression model was applied to CAP > 290 dB/m, and significant results were underlined for BMI (*p* value 0.000), oral diabetic therapy (*p* value 0.026), normal values of aminotransferase (*p* value 0.037), triglycerides (*p* value 0.010), and creatinine clearance value (*p* value 0.005); among them, just BMI value (0.006) and triglycerides (0.025) were confirmed by multivariate logistic regression model. Eventually, univariate regression model was used for FIB-4 > 2: we obtained significant results just for creatinine value (*p* value 0.045), a datum confirmed using the multivariate approach (*p* value 0.035). Statistical analyses were performed using SPSS 22.0 for Windows package. A *p* value lower than 0.05 was statistically significant.

## 6. Results

Among 382 T2D outpatients who consecutively attended the Metabolic Unit of the University Hospital of Messina from 1 January to 1 March 2021, 205 subjects [144 males (70%) and 61 females (30%), median age 64 years (range 40–84), median BMI 29.6 kg/m^2^ (range 20.4–49.6)] who met the inclusion criteria and consented to participate in this study were included (Table 1). The median diabetes disease duration was 11 years (range 1–42 years), and the median HbA1c value was 7.5% (range 4.9%–12.9%). Concerning T2D therapy, 87 (42.4%) subjects were on oral hypoglycemic agents (OHA) alone, 17 (8.3%) subjects were on only insulin, and 101 (49.2%) patients were in treatment with a combination of 2 or more drugs (including insulin); in particular, regarding innovative drugs, 60 patients (29.2%) were on Glucagon-like peptide-1 receptor agonist (GLP-1RAs) and 26 (12.6%) on SGLT2 inhibitors (SGLT2i).

A total of 125 also had also a previous diagnosis of arterial hypertension and 146 had a diagnosis of dyslipidemia. The amount of alcohol intake was more than 2 UI/die in 10% of the population.

**Figure 1:**
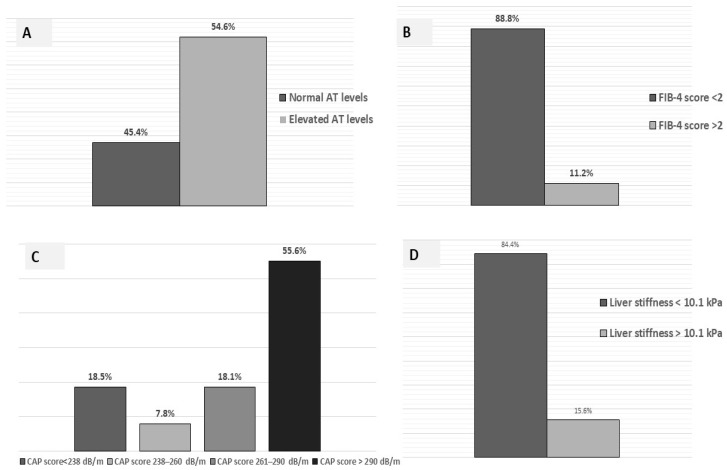
Cohort stratification according to AT (aminotransferase) levels (**A**), FIB-4 score values (**B**), CAP (Controlled attenuation parameter) value (**C**) and liver stiffness (**D**).

As shown in Figure 1, altered aminotransferase levels (AST and ALT > 40 U/L; GGT > 50 U/L) were detected in 112 (54.6%) (Figure 1A). The median FIB-4 score was 1.5 (range 0.00–12.76), and 11.2% of subjects had FIB-4 scores > 2 (Figure 1B). When patients were stratified into 4 groups on the basis of the FIB-4 value (<1.3; 1.3–2.0; 2.1–2.7; <2.7), 98 (47.8%) of them showed FIB-4 values < 1.3 (fibrosis excluded), 69 (33.6%) had FIB-4 value between 1.3–2.0 and 23 (11.2%) had FIB-4 value between 2.0 and 2.7, and 15 (7.3%) had a FIB-4 score value > 2.67 (high predictive for advanced fibrosis).

According to this study design, all patients underwent liver stiffness and CAP evaluation by FibroScan, showing a median value of 8.4 kPa (range 4.1 kPa–32.2 kPa) and 302 dB/m (range 103 dB/m–400 dB/m), respectively. In particular, 114 subjects (55.6%) had CAP values > 290 dB/m (severe steatosis) (Figure 1C), and 32 subjects (15.6%) had a liver stiffness value > 10.1 Kpa (severe fibrosis) (Figure 1D)

At univariate logistic regression analysis, liver stiffness value > 10.1 kPa (Table 2) were significantly associated with BMI (*p* = 0.000—Exp B 1.172% C.I.: 1.088–1.264), HbA1c (*p* = 0.002, Exp B 1.468; 95% C.I.: 1.146–1.881), and creatinine clearance values (*p* value 0.011, expB 1.013, C.I.: 1.003–1.023); these results were confirmed at multivariate logistic regression analysis with the exception of the creatinine clearance values: BMI (*p* value 0.001, Exp B 1.172, C.I.: 1.070–1.283), HbA1c (*p* value 0.010, Exp B 1.443, C.I.: 1.092–1.906).

At univariate logistic regression analysis, BMI values (*p* value 0.000, Exp B 1.143, C.I.: 1.074–1.216), oral diabetes therapy (*p* value 0.026, Exp B 2.773, C.I.: 1.129–6.815), AT values (*p* value 0.037, Exp B 1.817, C.I.: 1.038–3.181), triglycerides (*p* value 0.010, Exp B 1.005, C.I.: 1.001–1.009), and creatinine clearance (*p* value 0.005, Exp B 1.013, C.I.: 1.004–1.023) were associated with CAP score values > 290 dB/m (Table 3). At multivariate logistic regression analysis, BMI (*p* value 0.006, Exp B 1.112, C.I.: 1.031–1.199), and triglycerides (*p* value 0.025, Exp B 1.005, C.I.: 1.001–1.009) values were significant predictors of hepatic steatosis.

Finally, FIB-4 values (Table 4) were considered for both univariate and multivariate logistic regression models: at univariate logistic regression analysis, creatinine value (*p* value 0.045, Exp B 3.492, C.I.: 1.031–11.829) was identified; this variable was confirmed by multivariate logistic regression model (*p* value 0.035, Exp B 3.815, C.I.: 1.102–13.202). Notably, no significant associations at either univariate or multivariate analysis for liver stiffness, CAP and FIB-4 were noted for any hypoglycemic drug class, with the exception of the univariate association between treatment with oral hypoglycemic agents and CAP score.

## 7. Discussion

NAFLD affects ~30% of the general population [35] in developed countries, but it may be diagnosed in up to 70% of patients with T2D, with a higher risk of developing more severe forms and worse outcomes [36]. Despite such evidence, there is a lack of clear information on when and how to screen T2D outpatients in routine clinical practice.

In this study, in accordance with current Guidelines [13], we applied non-invasive methods in order to screen all consecutive T2D patients attending a single diabetes outpatient clinic after excluding other causes of liver disease.

In particular, the FIB-4 score was calculated to identify T2D patients with potentially metabolic liver disease, and a combination of US and transient elastography with CAP was performed in order to identify more severe forms and to address patients to further liver workouts, including liver biopsy, when necessary. Overall, our study showed that 55.6% of our T2D patients had unknown severe NAFLD based on CAP values. In these patients, the high risk of fibrosis should be considered to refer them to the correct clinical management.

Moreover, a serious warning came from our study since 23.9% of screened T2D patients had clinically meaningful liver harm, with a FIB-4 score > 2 and/or stiffness > 10.1 kPa. Similar results have been shown in a small study conducted in Morocco [37], where non-invasive hepatic biomarkers indicated the presence of significant fibrosis in 18.3% of the studied population.

The high prevalence of fibrosis observed in T2D outpatients without any known liver disease, and regularly attending a diabetes visit is particularly alarming since the presence of fibrosis has been associated with a higher risk of cirrhosis and mortality [38]. In addition, our data showed that obesity and poor glucose control were independent predictors of liver stiffness, and this information may be useful to individuate T2D individuals at higher fibrosis risk.

Although the pathophysiological mechanisms underlying the occurrence of liver fibrosis in patients with T2D are not fully identified yet, our data point to a relevant role for obesity and its corollaries in terms of insulin resistance, clarifying which patients should be considered at higher risk of the more severe forms of NAFLD.

NAFLD is considered the hepatic manifestation of visceral obesity and metabolic syndrome [39], and obesity and dyslipidemia have been proven to be involved in the initiation and progression of NAFLD in patients with T2D [40,41]. Moreover, NAFLD and T2D have common physio-pathological pathways represented by insulin resistance (IR), oxidative stress, systemic inflammation, and excessive production of free fatty acids (FFA) [42,43]. IR is considered the principal link between NAFLD and T2D for its effects on lipid metabolism [43], determining a deposit of lipid droplets in hepatocytes, which promote hepatocellular ballooning and lobular inflammation up to NASH development [44,45]. In line with this pathogenetic hypothesis, in our study, CAP levels > 290 dB/m were related to high BMI and triglycerides values, representing the triglycerides’ accumulation > 5% of liver weight as the basis for NAFLD.

Moreover, our results showed that a higher creatinine value was associated with a FIB-4 score value > 2. High creatinine values are associated with a 3.8 times greater risk of having a FIB-4 value > 2, suggesting liver fibrosis. These results are in accordance with recent studies supporting a relationship between chronic kidney disease (CKD) and the prevalence and severity of NAFLD [46,47]. Although several mechanisms have been proposed to justify this correlation, including metabolic syndrome, fructose and uric acid accumulation, oxidative stress, intestinal dysbiosis, platelet activation, and genetic predispositions [47,48], the pathogenetic link between NAFLD and microvascular complications in T2D patients still needs to be further investigated.

Contrariwise, AST and ALT values did not enter the predictive model. Thus, it is important to underline that although ALT levels still represent the most used laboratory parameter to screen for NAFLD in routine clinical practice, they cannot be considered an optimal marker to predict NASH and advanced fibrosis, showing poor sensitivity in early NAFLD diagnosis and prognosis [49]. On the other hand, the interplay between T2D and liver damage is not limited to NAFLD [50].

In line with the recent literature data, our study also underlines the importance of using a combination of non-invasive methods in order to screen T2D patients for NAFLD and its more severe forms in the “real world setting” of diabetes clinics. Considering the alarming prevalence of the severe forms of liver disease among patients with T2D unaware of the liver damage, the effective screening of T2D patients with non-invasive methods may help to identify individuals at high risk of advanced liver disease. Additionally, considering the increased risk of cardiovascular events associated with NAFLD, this systematic screening may help to prevent MACE in T2D patients [51].

Besides the pathogenetic background, the finding of a higher risk of liver fibrosis in unaware T2D obese patients should prompt physicians to investigate liver damage in these patients, also in light of their correct management. Thus, although no medical treatment has been currently authorized for the management of NAFLD in patients with T2D, a growing body of evidence points to the beneficial effects of insulin-sensitivity drugs, such as pioglitazone, metformin, GLP-1RAs, and SGLT2i, irrespective of their beneficial effect on body weight [52,53,54]. In particular, a recent meta-analysis showed that treatment with GLP-1RAs was associated with significant reductions in the absolute percentage of liver fat content, serum liver enzyme levels, and a greater histological resolution of NASH without worsening liver fibrosis compared to placebo or reference therapies, indicating the great potential of this class of drugs for the treatment of T2D patients with NAFLD [55]. However, when we verified the impact of these classes of hypoglycemic drugs on CAP, liver stiffness, and FIB-4 score, none showed significant associations either at univariate or multivariate regression analysis. The relatively small sample size, especially when considering the single drug classes, may likely have played a role in these results.

Several limitations should be acknowledged when interpreting our results. The cross-sectional study design impedes assessing causality, and the small sample size and the monocentric recruitment of patients may partly prevent the generalizability of our results to other study populations. The systematic screening approach by using a combination of non-invasive methods, the unselected T2D population screened in the setting of an outpatient diabetes clinic, the careful exclusion of potential causes of liver disease, including unknown virus hepatitis infection, and the continued follow-up of patients for their future evaluation should conversely be acknowledged among the strengths of this study.

Currently, a hint for future research is represented by the assessment of a possible association between liver disease progression and T2D complications, including both micro- and macro-vascular ones. Additionally, after the implementation of the sample, further stratifications of the study population could be made according to the prescribed therapies, with special attention to GLP1-RAs that represent the future of NAFLD treatment.

## 8. Conclusions

In conclusion, liver fibrosis is a frequent finding in T2D outpatients without a known history of liver disease, especially those with obesity, hypertriglyceridemia, worse glycemic control, and high creatinine levels. The use of a combination of non-invasive methods should be implemented in routine clinical practice in order to identify high-risk subjects and referred them to the appropriate management.

## Figures and Tables

**Table 1:** Demographic, clinical, biochemical and instrumental data of 205 patients stratified according to aminotransaminases (AT) levels.

	All T2D Subjects	Normal AT Levels	Elevated AT Levels	*p*-Value
N. (%)	205	93 (45.4)	112 (54.6)	-
Age (years)	64 (40–84)	65 (44–88)	62 (41–88)	0.05
Male n. (%)	144 (70.2)	61 (65.5)	83 (74.1)	0.13
BMI (kg/m^2^)	29.9 (20.4–49.6)	29.3 (21.2–45.4)	30.5 (21.2–45.9)	0.02
T2D duration (years)	11.1 (1–42)	11.6 (1–41)	10.7 (1–42)	0.5
Total Cholesterol (mg/dL)	166 (91–327)	161.6 (79–327)	169.9 (93–277)	0.10
LDL-cholesterol (mg/dL)	89 (42–190)	86.1 (18–192)	91.9 (11–210)	0.12
HDL-cholesterol (mg/dL)	47 (24–152)	48 (25–82)	45.6 (12–78)	0.38
Triglycerides (mg/dL)	158 (52–757)	141.4 (44–349)	172.5(20–662)	0.11
Creatinine (mg/dL)	1 (0.5–2.9)	0.9 (0.5–1.89)	1 (0.5–2.09)	0.21
Creatinine Clearance (mL/min/1.73 m^2^)	85 (26.6–110.8)	79.9 (19.8–184)	89.2 (28.2–202.6)	0.42
Platelet count (×10^3^/µL)	228 (139–613)	236 (145–613)	222 (134–386)	0.54
FIB-4 score	1.5 (0.8–4.25)	1.3 (0.5–3.85)	1.6 (0.00–3.41)	0.002
CAP (dB/m)	302 (103–400)	288.6 (103–400)	298.6 (124–400)	0.03
Stiffness (kPa)	8.4 (4.1–32.2)	6.9 (2.5–28.5)	9.3 (2.6–62.9)	0.000
Hypertension n. (%)	125 (60.9)	68 (72.3)	57 (50.9)	0.086
Lipid-lowering therapy n. (%)	146 (71.2)	69 (72.4)	77 (68.7)	0.28
Alcohol intake (>2 U/die) n. (%)	21 (10.2)	3 (3.2)	18 (16.1)	0.004
Insulin therapy n. (%)	69 (33.6)	33 (33.5)	36 (32.1)	0.61
Oral hypoglycemic agents n. (%)	181 (88.2)	81 (87.1)	100 (89.3)	0.63
GLP1-Ras n. (%)	60 (29.2)	22 (23.6)	38 (33.9)	0.06

Data are n, % and median and range. Abbreviations: AT (Aminotransferase), BMI (Body mass index), CAP (Controlled attenuation parameter), T2D (type 2 diabetes mellitus), GLP1-Ras (Glucagon-like peptide 1—receptor agonist). AST and ALT ≤ 40 UI/L, and γ-GT ≤ 50 UI/L were considered normal values. Study subjects were in treatment with insulin and/or oral hypoglycemic agents and/or GLP-1RAs or a combination of these drugs.

**Table 2:** Univariate and Multivariate regression models for severity of fibrosis (FibroScan > 10.1 kPa).

	Univariate Model			Multivariate Model		
Variables	OR	95% C.I.	*p*-Value	OR	95% C.I.	*p*-Value
Sex	0.876	0.399–1.925	0.742			
Age	0.974	0.937–1.013	0.189			
BMI	1.172	1.088–1.264	0.000	1.172	1.070–1.283	0.001
T2D duration	0.954	0.907–1.004	0.069			
Insulin therapy	1.394	0.659–2.947	0.385			
Oral diabetes therapy	2.453	0.550–10.947	0.240			
HbA1c (%)	1.468	1.146–1.881	0.002	1.443	1.092–1.906	0.010
AT	2.010	0.927–4.360	0.77			
PLT	1.004	0.998–1.010	0.167			
Total Cholesterol	1.000	0.990–1.009	0.918			
Triglycerides	0.999	0.995–1.004	0.712			
HDL-C	0.981	0.947–1.017	0.304			
LDL-C	1.001	0.991–1.010	0.888			
Creatinine	0.720	0.185–2.805	0.636			
Creatinine clearance	1.013	1.003–1.023	0.011	0.998	0.986–1.011	0.779
Hypertension	1.161	0.531–2.536	0.708			
Lipid-lowering therapy	0.829	0.376–1.828	0.643			

Abbreviations: BMI (Body mass index), AT (aminotransferase), PLT (Platelets), HbA1c (Glycated Hemoglobin), HDL-C (HDL Cholesterol), LDL-C (LDL Cholesterol), T2D (type 2 diabetes mellitus), AST and ALT ≤ 40 UI/L, and γ-GT ≤ 50 UI/L were considered normal values.

**Table 3:** Univariate and Multivariate regression models for liver steatosis (CAP > 290 dB/m).

	Univariate Model			Multivariate Model		
Variables	OR	95% C.I.	*p*-Value	OR	95% C.I.	*p*-Value
Age	0.993	0.966–1.021	0.663			
Sex	1.237	0.676–2.264	0.490			
BMI (kg/m^2^)	1.143	1.074–1.216	0.000	1.112	1.031–1.199	0.006
T2D duration	0.983	0.951–1.016	0.308			
Insulin therapy	0.910	0.508–1.630	0.750			
Oral diabetes therapy	2.773	1.129–6.815	0.026	2.408	0.925–6.271	0.072
HbA1c (%)	1.001	0.823–1.217	0.995			
AT	1.817	1.038–3.181	0.37	1.429	0.771–2.649	0.257
PLT	1.000	0.995–1.005	0.968			
Total Cholesterol	1.003	0.996–1.010	0.385			
Triglycerides	1.005	1.001–1.009	0.10	1.005	1.001–1.009	0.025
HDL-C	0.990	0.965–1.016	0.446			
LDL-C	0.999	0.991–1.006	0.695			
Creatinine	0.721	0.301–2.295	0.721			
Creatinine clearance	1.013	1.004–1.023	0.005	1.003	0.992–1.015	0.584
Hypertension	1.069	0.594–1.925	0.824			
Lipid-lowering therapy	0.643	0.343–1.208	0.170			

Abbreviations: BMI (Body mass index), AT (aminotransferase), PLT (Platelets), HbA1c (Glycated Hemoglobin), HDL-C (HDL Cholesterol), LDL-C (LDL Cholesterol), CAP (Controlled attenuation parameter), T2D (type 2 diabetes mellitus), AST and ALT ≤ 40 UI/L, and γ-GT ≤ 50 UI/L were considered normal values.

**Table 4:** Univariate and multivariate regression models for FIB-4 > 2.

	Univariate Models			Multivariate Model		
Variables	OR	95% C.I.	*p*-Value	OR	95% C.I.	*p*-Value
Sex	2.426	0.955–6.162	0.062			
T2D duration	1.001	0.959–1.044	0.959			
Insulin therapy	0.802	0.370–1.739	0.577			
Oral diabetes therapy	0.620	0.228–1.688	0.350			
HbA1c (%)	0.905	0.696–1.177	0.457			
Total Cholesterol	0.994	0.985–1.004	0.249			
Triglycerides	0.999	0.995–1.004	0.759			
HDL-C	1.007	0.975–1.041	0.656			
LDL-C	0.992	0.981–1.002	0.133			
Creatinine	3.492	1.031–11.829	0.045	3.815	1.102–13.202	0.035
Creatinine clearance	0.987	0.974–1.000	0.044			
Hypertension	0.714	0.343–1.484	0.366			
Lipid-lowering therapy	1.075	0.483–2.394	0.859			

Abbreviations: HbA1c (Glycated Hemoglobin), HDL-C (HDL Cholesterol), LDL-C (LDL Cholesterol), T2D (type 2 diabetes mellitus), AST and ALT ≤ 40 UI/L, and γ-GT ≤ 50 UI/L were considered normal values.

## Data Availability

Data are unavaiable due to privacy.
